# Different Concentrations of Doxycycline in Swine Manure Affect the Microbiome and Degradation of Doxycycline Residue in Soil

**DOI:** 10.3389/fmicb.2018.03129

**Published:** 2018-12-19

**Authors:** Qiufan Yan, Xiaoyang Li, Baohua Ma, Yongde Zou, Yan Wang, Xindi Liao, Junboo Liang, Jiandui Mi, Yinbao Wu

**Affiliations:** ^1^College of Animal Science, National Engineering Research Center for Breeding Swine Industry, South China Agricultural University, Guangzhou, China; ^2^Nanhai Entry-Exit Inspection and Quarantine Bureau, Foshan, China; ^3^Guangdong Enterprise Lab of Healthy Animal Husbandry and Environment Control, Yunfu, China; ^4^Ministry of Agriculture Key Laboratory of Tropical Agricultural Environment, South China Agricultural University, Guangzhou, China; ^5^Guangdong Provincial Key Laboratory of Agro-Animal Genomics and Molecular Breeding, South China Agriculture University, Guangzhou, China; ^6^Laboratory of Animal Production, Institute of Tropical Agriculture, University Putra Malaysia, Serdang, Malaysia; ^7^Laboratory of Molecular Biomedicine, Institute of Bioscience, University Putra Malaysia, Serdang, Malaysia

**Keywords:** doxycycline degradation, soil microbiome, interaction effect, resistance gene, swine manure

## Abstract

Antibiotic residues that enter the soil through swine manure could disturb the number, community structure and functions of microbiota which could also degrade antibiotics in soil. Five different concentrations of doxycycline (DOX) incorporated into swine manure were added to soil to explore the effects of DOX on microbiota in soil and degradation itself. The results showed that the soil microbiome evolved an adaptation to the soil containing DOX by generating resistance genes. Moreover, some of the organisms within the soil microbiome played crucial roles in the degradation of DOX. The average degradation half-life of DOX in non-sterile groups was 13.85 ± 0.45 days, which was significantly shorter than the 29.26 ± 0.98 days in the group with sterilized soil (*P* < 0.01), indicating that the soil microbiome promoted DOX degradation. DOX addition affected the number of tetracycline resistance genes, depending on the type of gene and the DOX concentration. Among these genes, *tet*A, *tet*M, *tet*W, and *tet*X had significantly higher copy numbers when the concentration of DOX was higher. In contrast, a lower concentration of DOX had an inhibitory effect on *tet*G. At the same time, the microbial compositions were affected by the initial concentration of DOX and the different experimental periods. The soil chemical indicators also affected the microbial diversity changes, mainly because some microorganisms could survive in adversity and become dominant bacterial groups, such as the genera *Vagococcus* and *Enterococcus* (which were associated with electrical conductivity) and *Caldicoprobacter* spp. (which were positively correlated with pH). Our study mainly revealed soil microbiota and DOX degradation answered differently under variable concentrations of DOX mixed with swine manure in soil.

## Introduction

Tetracycline (TC) antibiotics are usually used for maintain animal health and curing livestock and poultry disease. which is important for animal husbandry. As a member of the TCs and because of superior properties that include high bioavailability, shorter half-life and stronger antibacterial activity, doxycycline (DOX) is widely used in aquaculture production. However, just a few veterinary antibiotics can be absorbed by animals. As much as 30–90% veterinary antibiotics entered to soil and surface water via excreta in prototype or metabolites (Jjemba, 2006; Daghrir and Drogui, 2013; Li et al., 2015). The proportion of DOX excreted by the rearing layers from the intake of feed containing different concentrations of DOX is 82.67, 94.39, 95.72%, respectively (Peng et al., 2016). Several studies have shown the detection of DOX residues in environmental media, such as animal manure, soil, and water. Wu et al. (2010) collected soil samples from Beijing, Tianjin, etc., and then DOX residues at a concentration of 0.16–5.01 μg/kg were detected. Ho et al. (2014) reported that residual levels of DOX in the soil of Malaysian farmlands varied from 60 to 720 μg/kg. Furthermore, Lindberg et al. (2005) detected antibiotic residues in a wastewater treatment tank in Sweden, in which the DOX concentration was 1.5 mg/kg.

Currently, environmental issues are attracting increasing attention. Residues of antibiotics may have an effect on the normal physiological metabolism of lives on earth, affecting the ecosystem function through the activities of degradation, adsorption, and migration. The microbial community structure can be changed in individual genes or species and may also be adjusted by the proportion of microorganisms (Ding and He, 2010). Huang et al. (2015) found that *Bacteroidetes* and *Proteobacteria* had a greater abundance in sludge with increased TC concentrations. The total number of antibiotic resistance bacteria (ARB) in soil increased because of the addition of TC (Sengelov et al., 2003). In addition, the copies of fungi and bacteria in soil would increase when added oxytetracycline (one of the TCs) to soil (Colinas et al., 1994). Antibiotics remained in soil will not only change the microbiome composition and number in soil but also inhibit the function of microorganisms. Chen et al. (2014) found that oxytetracycline metabolized *in vivo* can significantly increase the activity of urease and catalase in soil and reduce the activity of alkaline phosphatase after the animals’ urine and manure enter into the soil. However, Yang et al. (2009) added 10–30 mg/kg oxytetracycline to soil, then found that the phosphatase activity in the soil decreased from 80.8 to 41.3% but that there was no significant effect on catalase and urease activity. This difference may result in different methods of adding oxytetracycline and different concentrations of oxytetracycline. Therefore, our research has explored microbial function under different DOX concentrations.

Several studies have mainly conducted the degradation of antibiotics in the environment through microbial degradation. Microorganisms can produce metabolic enzymes and other substances in a specific environment that will modify the structure of an antibiotic and directly or indirectly deactivate it. Chang et al. (2014) found the biodegradation of TCs in was 92.90–100%, while the TCs remained at 6.80–8.50% in the sludge. Wu et al. (2009) found that the concentration of DOX after sterilization changed little during the entire experimental period and that the degradation rate of DOX in the non-sterilized treatment was much higher. In recent years, some scholars have also isolated strains that can degrade certain antibiotics and use degradative enzymes to degrade environmental antibiotics, which can provide more efficient and reliable microbiological methods (Migliore et al., 2012).

Environmental antibiotic residues can change microbial community structure, number and function; however, microorganisms are not only inhibited or killed by antibiotics but also produce antibiotic resistance genes (ARGs), so they can survive. In recent years, ARGs were detected in animal excreta, sewage, and soil (Peak et al., 2007; Zhang et al., 2009). Chee-Sanford et al. (2009) detected eight tetracycline resistance genes that encoding ribosome protective proteins in a septic tank near a swine farm. Peak et al. (2007) found that *tet*Q, *tet*W, *tet*O, *tet*M, *tet*B and *tet*L were significantly higher than a United States cattle farm wastewater pool. Therefore, we investigated ARGs to explore the interaction between DOX and soil microorganisms.

Comparing the different changes between sterilization and non-sterile treatments is a common method for studying microbial degradation (Chen et al., 2014). However, most of these experimental designs are based on a single substrate (soil or manure), which may not represent the real environmental conditions, which are generally a mixture of manure and soil. However, whether microbial degradation behavior will be influenced by different TC concentrations remains unknown. Therefore, in this study, DOX was chosen as an object to clarify the microbial degradation behavior in soil mixed with swine manure under different concentrations of DOX.

## Materials and Methods

### Preparation of Experimental Materials

In the initial stage of the experiment, we selected 60 fattening pigs with good conditions and weighing approximately 30 kg for DOX feeding. Feed method was showed in Supplementary Material. During this period, we collected about 2 kg of excrement manure in each group twice a day. The moisture content of collected manure was 68% on average. And the characteristics of samples were showed in Supplementary Table S1.

Soil without fertilizing with organic fertilizer for 180 days was collected in Guangzhou, South China. We found that the soil was free of DOX by LC-MS analysis. The characteristics of the soil samples were shown as follows: maximum field moisture content capacity: 52.62%, electrical conductivity (EC): 1.07 μs/cm, pH: 6.18, total carbon (TC): 10.86 g/kg, total nitrogen (TN): 1.03 g/kg.

### Experimental Design

After finishing the previous experiment, the concentrations of DOX in collected manure were detected for subsequent experiment. The subsequent experiments were completely randomized and were divided into two treatments: (A) a non-sterile treatment for the soil-mixed manure and (B) a sterilization treatment for the soil-mixed manure. The method of sterilizing involved chemical sterilization by adding NaN_3_ to the soil at 0.1% addition.

According to the detection results for the DOX concentration in the collected swine manure, five groups were set up in each treatment, and the manure was mixed with soil at a ratio of 5:95 based on dry weight. By detecting the DOX concentration in the above mixture, the initial DOX concentrations were 0, 4.4, 8.0, 10.9, and 13.2 μg/g, respectively. The experimental group and the initial concentrations of DOX are listed in Supplementary Table S2.

Each treatment has three replicates. The soil culture method was based on Chen et al. (2014), which was showed in Supplementary Material. Samples were collected from each replicate in 0, 1, 3, 8, 15, 21, 26, and 33 days. These samples were used to determine the DOX contents, soil moisture content, pH, electric conductivity, organic matter, and total nitrogen and catalase.

### Detection of DOX

About 2 g of soil sample was sampled to extract DOX. The method for extracting DOX and LC-MS conditions were based on Ren et al. (2017), which was showed in Supplementary Material. The identification and quantification of DOX were summarized in Supplementary Table S3. The recovery rate of soil supplemented with DOX was 82%, and the limit of detection and quantification are 0.02 and 0.06 mg ⋅ kg^-1^, respectively.

### Isolation of Total DNA

EZNATM Soil DNA extraction kit (OMEGA) was used to extract sample DNA. DNA products were sent for the high-throughput sequencing using Miseq platform and used for real-time PCR.

### Real-Time Quantitative PCR

Both the chemical and equipment used for qPCR were obtained from Bio-Rad (United States). The primers were used in accordance with a previous study (Supplementary Material, Aminov et al., 2001; Yang and Doong, 2008). In addition, the temperature procedure of the qPCR and the details of the thermocycling protocol were shown in the Supplementary Tables S4, S5.

### Information Sequencing and Analysis Process

Sequencing was performed according to the standard library of Honor Technology in Beijing, and the main data on the MiSeq platform was filtered for analysis. Raw data obtained by sequencing have a certain proportion of dirty data. To make the information analysis result more accurate and reliable, the original data were first spliced and filtered to obtain clean data. Then, based on clean data, OTU clustering and species classification analysis were performed, and OTU and species annotations were combined to obtain the basic analysis results of the OTUs and the classification spectrum of each sample. Then, the abundance and diversity index of OTUs were analyzed, and at the same time, a statistical analysis of the species structure was performed on the species annotation at each level of classification. Finally, on the basis of the above analysis, a series of cluster analyses based on OTUs, species composition, PCoA and PCA, CCA and RAD were compared and analyzed, the differences in species composition between samples were explored, and the association analysis was performed in combination with environmental factors.

### Statistical Analyses

We use SPSS 18.0 to analyze the data obtained from the experiment, and Tukey tests were used to significance analysis. *P-*value < 0.05 meaning there are statistical significance. Part of the data used SAS to investigate the L, Q and C effects under GLM model.

## Results

### DOX Degradation in Soil

**FIGURE 1:**
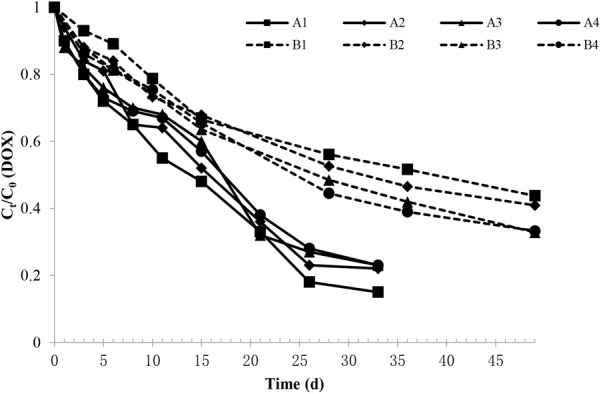
Changes in the DOX relative content in each experimental group. The A1, A2, A3, and A4 groups were all non-sterile test groups, and the B1, B2, B3, and B4 groups were all sterilization test groups, which were pig manure + blank soil groups with initial DOX concentrations of 4.4, 8.0, 10.9, and 13.2 mg/kg, respectively.

The DOX degradation in the soil during the experimental period in each group is shown in Figure 1. The figure shows that the DOX concentration in soil degraded faster during the early period, and as the experiment progressed, the speed of degradation gradually slowed. By comparing with the sterilization group, the degradation rate in the non-sterile group was more rapid, showing that on the 33rd day, the DOX degradation rate in the non-sterile group was 77–85%; on the 49th day, the DOX degradation rate was only approximately 43–65% in the sterilization group.

Doxycycline was fitted with concentration curves at different sampling times, and the DOX under different treatment conditions were in line with the level of degradation in the soil dynamics equation *C* = *C*_0_e^-^*^kt^*, which C represent for DOX concentration (mg/kg), *C*_0_ represent for the initial DOX concentration (mg/kg), *k* represent for the rate constant (d^-1^) and *t* represent for the time in days. Upon inspection, the fitting was significant (*F* > 0.05), and the correlation coefficient (*R*^2^) was 0.94. Equations can be obtained by fitting the calculated different initial concentrations in the soil degradation half-life of DOX, as shown in Table 1.

**Table 1:** Degradation kinetic equation and half-life of DOX in each experimental treatment.

Item	Treatment	The initial concentration of doxycycline (mg/kg)				*P-*value ^1^		
		4.4	8.0	10.9	13.2	L	Q	C
First-order kinetic equation	A	*C* = 4.41e^–0.057t^	*C* = 7.95e^–0.049t^	*C* = 10.88e^–0.047t^	*C* = 13.20e^–0.046t^	–	–	–
	B	*C* = 4.41e^–0.021t^	*C* = 7.95e^–0.022t^	*C* = 10.88e^–0.026t^	*C* = 13.20e^–0.027t^			
Half-life (d)	A	12.21 ± 0.93^Ba^	14.44 ± 0.68^Bb^	15.42 ± 1.43^Bc^	15.43 ± 0.85^Bc^	<0.001	<0.001	<0.001
	B	33.63 ± 0.73^Aa^	30.69 ± 0.46^Ab^	26.78 ± 0.45^Ac^	25.91 ± 2.16^Ac^	<0.001^X^	<0.001^X^	<0.001^X^
*R*^2^	A	0.98 ± 0.009	0.98 ± 0.006	0.93 ± 0.250	0.95 ± 0.164	<0.001	<0.001	<0.001
	B	0.96 ± 0.169	0.94 ± 0.208	0.97 ± 0.002	0.97 ± 0.033	<0.001^X^	<0.001^X^	<0.001^X^

*^a,b^ Within a column, means without a common superscript differ (*P* < 0.05). ^x^*P*-value for interaction of treatment effect with initial concentration of DOX.*

Analysis of variance showed that the sterilization DOX degradation half-life was significantly longer than that those non-sterile groups (*P* < 0.01). It was noted that DOX biodegradation in soil played an important role and that the DOX degradation time in sterilization soil was significantly extended.

For the same treatment in groups with different DOX concentrations, *t*-test results showed that DOX degradation half-life in A1 was significantly shorter than that in A2 (*P* < 0.01) and that the DOX degradation half-life was not significantly different among the rest treatments (*P* > 0.05). DOX degradation rate under the influence of its initial concentration in the soil showed that if the initial concentration is lower, the degradation rate is faster. At higher initial concentrations of DOX, the inhibitory effect on the soil microbial degradation of DOX was stronger, and thus, the degradation was slower.

With sterilization, the DOX degradation half-life was longer in B1 than that in B2 (*P* < 0.05), and the B1 and B2 DOX degradation half-lives were significantly longer than B3 and B4 groups (*P* < 0.01). The DOX degradation rate in the sterilization soil was influenced by DOX concentration. The initial concentration was lower, and the degradation was slower.

The L, Q and C effects were analyzed to investigated the interaction effect between the initial concentrations of DOX and treatment. The results showed that both the treatment and the initial concentration of DOX had significant effects on *R*^2^ and half-life of DOX degradation.

### Changes in Tetracycline Resistance Genes in Soil

During the experiment, the change trends of *tet*A, *tet*G, *tet*M, *tet*W, and *tet*X in the non-sterile treatment are shown in Table 2. Analysis of variance showed that the copies of *tet*M, *tet*W, *tet*A, *tet*G, and *tet*X in the CK were lower than other treatments (*P* < 0.05). The number of *tet*A genes of CK group remained stable during the experiment. As the experiment progressed, the number of *tet*A genes in the A0–A4 group first increased and then decreased. In the experimental groups with different concentrations of DOX, there is a significant difference in the number of tetA genes. The results showed that the total content of *tet*A in the A0 group was the highest, which was higher than A1–A4 groups (*P* < 0.05).

**Table 2:** Changes in the copies of different tetracycline resistance genes in soil.

Treatment_day	The total number of ARGs log (*copies/g* DM)				
	*tet*A	*tet*G	*tet*M	*tet*W	*tet*X
CK_0	2.58	1.08*	3.70**	2.83**	2.63*
A0_0	3.03	2.15	5.58	6.02	3.80
A1_0	3.98*	2.01	6.07	5.88	3.27
A2_0	2.67	1.41	5.81	5.90	3.53
A3_0	3.49*	1.27	6.37	6.20	4.07
A4_0	2.83	1.88	5.80	6.26	4.10
CK_8	3.09 *	1.91 *	3.82 **	2.37 **	3.28 **
A0_8	4.60 *	4.45	6.99	5.46	5.96
A1_8	4.05	3.39 *	7.18	5.71	6.24
A2_8	3.85	4.58	7.39	5.70	6.23
A3_8	3.68	4.20	7.41	5.52	6.50
A4_8	4.02	4.54	7.38	5.81	6.35
CK_15	2.67 *	2.36 *	4.05 **	3.26 **	2.82 **
A0_15	5.35 *	4.16	7.01	5.36	7.25
A1_15	3.87	2.85 *	7.35	5.41	6.52
A2_15	4.20	4.01	6.85	5.01	7.04
A3_15	4.04	4.45	6.98	5.39	6.23
A4_15	3.89	4.38	6.78	5.52	6.44
CK_22	2.78	2.66 *	4.15 **	2.54 **	3.40 **
A0_22	4.51 *	4.25	6.59	5.11	6.15
A1_22	3.27	3.81 *	6.96	5.12	5.58
A2_22	4.85 *	4.16	7.26	5.36	6.54
A3_22	3.73	4.47	6.78	5.04	5.29
A4_22	3.46	3.89	6.75	5.21	5.88
CK_33	2.65	2.29 *	3.81 **	2.92 **	2.90 **
A0_33	4.99 *	3.85	6.95	5.41	7.35
A1_33	4.16	4.83	7.40 *	5.41	7.11 *
A2_33	3.85	4.49	6.58	5.09	6.07
A3_33	4.55	4.88	6.20	5.00	5.96
A4_33	3.89	4.19	6.05	5.18	5.84

*^∗^ means difference at a level of *p* < 0.05; ^∗∗^ means difference at a level of *p* < 0.01.*

The total number of *tet*G in each group was basically the same at the beginning of the experiment. As the experiment progressed, the number of *tet*G in each group tended to be stable after the first increase. The number of *tet*G was significantly influenced by different concentrations of DOX. The results showed that the number of *tet*G in group A1 was the lowest (*P* < 0.05). The number of *tet*M in the CK group remained stable throughout the duration of the experiment. The copies of *tet*M in the other groups first increased and then decreased as the experiment progressed. The number of *tet*M was also significantly influenced by different concentrations of DOX. The number in group A1 was the highest and was significantly higher than the numbers in A0, A2, A3, and A4 (*P* < 0.05).

The number of *tet*W in CK fluctuated slightly during the experiment. The copies of *tet*W in the other groups showed a trend of decreasing gradually and finally stabilizing throughout the duration of experiment. On the 33rd day, the copies of *tet*W in the groups that were supplemented with pig manure dropped by an order of magnitude compared to those at the start of the experiment. The number of *tet*X in the CK group remained stable throughout the duration of the experiment. The copies of *tet*X in the other groups first increased, then decreased and then increased again. The number of *tet*W and *tet*X among the five groups has no different results (*P* > 0.05), which showed that DOX addition had little effect on the changes in the *tet*W and *tet*X content of soil microbes. However, ANOVA analysis of *tet*W and *tet*X number in each experimental group at the 33rd day showed that the *tet*W and *tet*X numbers in the experimental groups with different concentrations of DOX were significantly different and that the copy numbers of *tet*W and *tet*X in the A0 and A1 groups were the highest.

### DOX Effects on the Number of Microbial Communities

**FIGURE 2:**
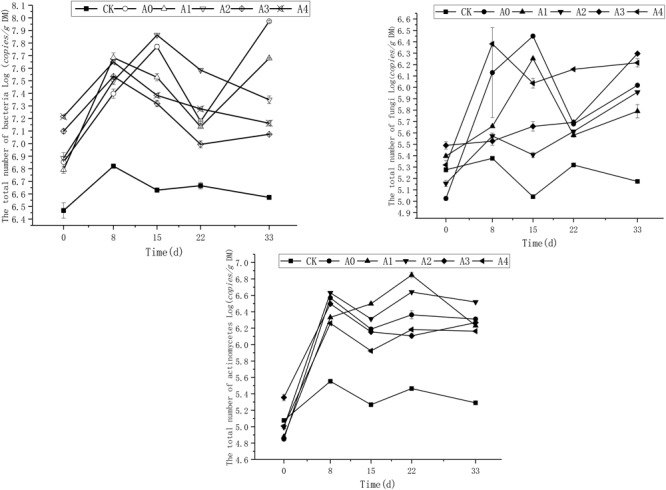
Trends in the total number of bacteria, fungi, and actinomycetes in the soil. The CK, A0, A1, A2, A3, and A4 groups were all non-sterile treatment groups, of which CK was a blank soil group; A0 was a blank pig manure + blank soil group; and A1, A2, A3, and A4 were pig manure + blank soil groups with initial DOX concentrations of 4.4, 8.0, 10.9, and 13.2 mg/kg, respectively.

The change of total number of bacteria, fungi, and actinomycetes in each experimental group under non-sterile treatment during the experiment are shown in Figure 2. As shown in the figure, the copies of bacteria in CK was basically stable during the experiment. The copies of soil bacteria in the other groups was generally consistent with the initial state. As the experiment progressed, the number of soil bacteria in A0 and A1 first increased, then decreased and finally increased again, while the bacteria in the other groups showed a tendency of first increasing, then decreasing and finally stabilizing. Variance analysis showed that the in the CK group, total number of bacteria was lower than other treatments (*P* < 0.05). These groups containing different concentrations of DOX kept stable (*P* > 0.05). However, on the 33rd day, there was difference in bacteria copies in these groups (*P* < 0.05). The results showed that the copies of soil bacteria in the A0 group without DOX was the highest and was significantly higher than the numbers in the A1–A4 groups (*P* < 0.05). The total numbers of bacteria of A2, A3, and A4 groups had no difference (*P* > 0.05).

Fungi population in CK group was relatively stable during the experiment, and the total number of soil fungi in each group was basically the same at the beginning of the experiment; as the experiment progressed, the number of soil fungi first increased, then dropped and then increased again. Variance analysis revealed that the total copies of fungi in CK was lower than other treatments (*P* < 0.05). The results showed that the number of total soil fungi in group A4 was the highest, which was significantly higher than A0–A3 (*P* < 0.05).

The total number of actinomycetes in CK was relatively stable during the experiment, and the total number of soil actinomycetes in the other groups was basically the same at the beginning of the experiment. As the experiment progressed, the total number of soil actinomycetes in the A1 group showed a gradual increase in the early period and finally decreased. In the other groups, this number first increased, then decreased, then increased again, and finally stabilized. Variance analysis showed that the total number of actinomycetes in CK was significantly lower than that other groups treated with pig manure (*P* < 0.05). The total number of actinomycetes in groups A1 and A2 was the highest, which was significantly higher than that in groups A0, A3, and A4 (*P* < 0.05).

### DOX Effects on the Diversity of the Bacterial Community Structure in Soil

Bacterial composition in CK clustered more closely to that of the other groups (Supplementary Figure S1). On the 0th day and 33rd day, groups A1–A4 were clustered. Among these groups, the low DOX concentration groups A0, A1, and A2 had similar trends on the 15th day and clustered together, while the high DOX concentration groups A3 and A4 did not aggregate with the A0, A1, and A2 groups. (Supplementary Figure S1).

**FIGURE 3:**
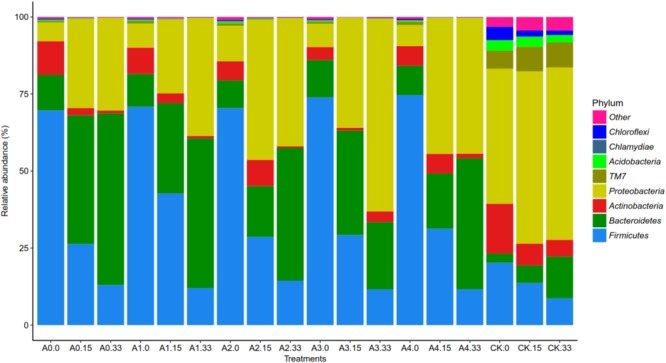
Changes in the relative abundance of phylum-level bacteria. Different colors represent different phylum-level species.

The highest frequency occurred when building the OTU species annotation of the sequence database (Supplementary Figure S2), which could be generated in phyla, classes, orders, families, and the relative abundance of species on the genus level histogram. Each group-level species composition of the phylum is shown in Figure 3. Overall, for many groups of soil bacteria, the mixture of pig manure with different initial concentrations of DOX yielded fewer bacterial species, and these mixtures of pig manure and DOX significantly reduced the abundance of soil bacteria. Levels of changes in the relative abundance of the species were tested at 0 days, with different initial concentrations after the addition of DOX and pig manure to the soil; in groups A0–A4, the *Firmicutes* and *Bacteroidetes* abundance increased, accounting for 69.7%, 74.6%, and 12.2%, respectively. The abundance of *Firmicutes* and *Bacteroidetes* in the CK groups increased by 20.2% and 3%, and in groups A0–A4, the *Proteobacteria* abundance decreased significantly to a proportion of 6.1–11.6%. The abundance of *Proteobacteria* in group CK was 43.8%; 15 days after the addition of pig manure to the treatment group, the relative abundance of *Firmicutes* decreased and that of *Proteobacteria* and *Bacteroidetes* increased dramatically. The DOX-treated test groups A1, A2, A3, and A4 treated with blank pig manure showed a smaller abundance of *Bacteroidetes* than group A0. The DOX-treated groups showed a greater abundance of *Proteobacteria* than group A0. DOX treatment suppressed the abundance of *Bacteroidetes* but increased the abundance of *Proteobacteria* compared with that at 15 days, and on the 33rd day of the above treatment, the effect became more obvious, showing that the addition of DOX significantly inhibited *Bacteroidetes* but increased the relative abundance of *Proteobacteria*.

**FIGURE 4:**
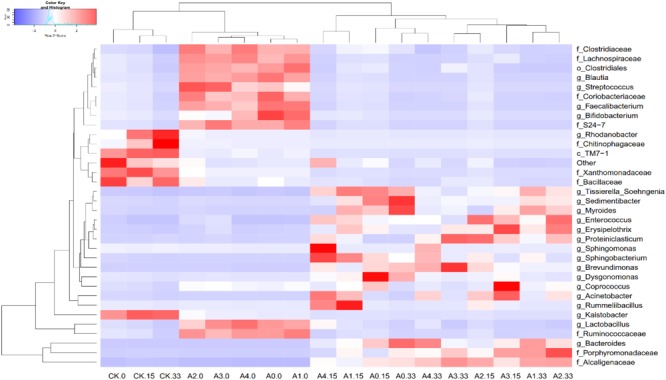
Changes in the relative abundances of predominant genera in different groups and on different days. Bacterial genera are color-coded according to their under- (red) or overrepresentation (blue) in the samples.

**FIGURE 5:**
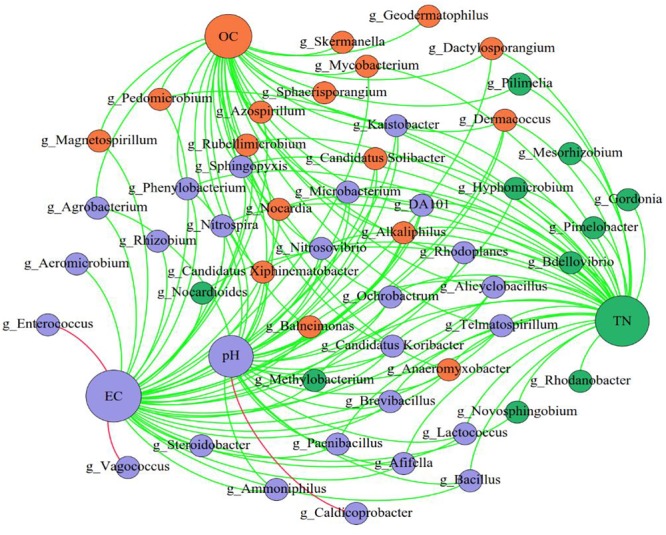
The network of genus and soil physical and chemical indicators. The nodes represent the predominant genera, and the size of each node is proportional to the degree (the number of connections). The edges represent strong and significantly positive (red) or negative (green) correlations between predominant genera and soil physical and chemical indicators. The nodes are colored based on the module structure.

By comparing the correlations among bacteria, we observed positive correlations between species, of which *Pedomierobium* spp. had the highest correlation with other bacteria (Supplementary Figure S3). At the genus level, in the CK group, the community structure of the microorganisms clustered over time, but the *Rhodanobacter* spp. increased significantly over time. On the 0th day, the experimental groups had no significant difference. On the 15th day, the microbial community structure of the high-DOX-concentration A4 was significantly higher than the low-DOX-concentration A1 and A0 groups, of which the *Sphingomonas* spp. was the most obvious. On the 33rd day, the microbial community structure of the experimental group showed a phenomenon in which the low-concentration group was higher than the high concentration group (Figure 4). To explore the mechanism of microbial community changes, we correlated microbial community diversity (general level) with soil physical and chemical indicators. The network diagram reveals a negative correlation between the level of soil organic matter, TN and microbial structure. In addition to Enterococcus spp., *Vagococcus* is positively correlated with EC, other indicators are negatively correlated with EC, and pH has a positive correlation only with *Caldicoprobacter* (Figure 5). It can be seen from the heat map that the correlation coefficient between the *Vagococcus* spp. and the EC is the highest, which is positively correlated, and the positive correlation coefficient between the genus *Caldicoprobacter* and pH is the highest, indicating that soil physicochemical indexes can affect the changes of soil microbial diversity (Supplementary Figure S4).

### Effects of Doxycycline on Soil Microbial Function

**FIGURE 6:**
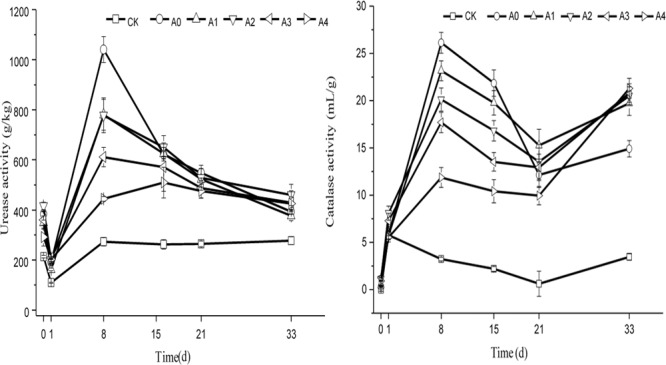
Changes in the catalase and urease activity in soil. The CK, A0, A1, A2, A3, and A4 groups were all non-sterile treatment groups, of which CK was a blank soil group; A0 was a blank pig manure + blank soil group; and A1, A2, A3, and A4 were pig manure + blank soil groups with initial DOX concentrations of 4.4, 8.0, 10.9, and 13.2 mg/kg, respectively.

The change trend of urease and catalase activity in various groups under non-sterile treatment is shown in Figure 6. At the beginning of the experiment, the soil urease activity of each experimental group was maintained at 200–400 g/kg. As the experiment progressed, the activity of urease in each group first decreased and then increased. On the 8th day, the urease activity reached its peak, and the urease content was maintained at 260–1,050 g/kg. The urease content then began to decline gradually and finally remained stable. At the end of the experiment, the urease content of each group was maintained at 250–500 g/kg. Variance analysis showed that urease activity in CK was lower than A0–A4 (*P* < 0.05), indicating that addition pig manure was conducive to increasing urease activity. The results showed that the soil urease activity was the highest in the A0 group, followed by that in A2, which was higher than A4 (*P* < 0.05).

Catalase activity in soil of each experimental group was very low, and its value was between 0 and 1.3 mL/g at the beginning of the experiment. As the experiment progressed, the soil catalase activity first increased and then decreased. The activity of catalase in the CK group changed more slowly than that in the other groups throughout the duration of the experiment. On the 33rd day, the catalase activity was maintained at 3.2–3.9 mL/g in the CK group, and the other activities remained between 14.2 and 20.2 mL/g. Variance analysis showed that catalase activity in CK was lower than other groups (*P* < 0.05). Those groups had no difference from each other (*P* > 0.05). However, on the 33rd day, the soil catalase activity significantly differed among these groups, which were supplemented with pig manure, showing that the soil catalase activity in A0 was the lowest, lower than A1–A4 (*P* < 0.05).

## Discussion

Currently, DOX has been widely used in animal husbandry and has been detected in feces, soil, water, and other environmental media. Because of its broad-spectrum antibacterial activity, its residues in environmental media may affect the number, community structure and function of microbial, thereby affecting the stability of the entire ecosystem. Moreover, soil microorganisms can also acquire antibiotic resistance through gene mutation or horizontal gene transfer (HGT) and then adapt to the effect of DOX. In the environment, antibiotics undergo a series of degradation reactions, including hydrolysis, photolysis, and biodegradation. Researchers have found that antibiotics in feces are degraded mainly through photolysis in soils. All three degradation reactions are important in water.

By studying the behavior of DOX degradation in sterilization soil and non-sterile soil, we found that microbial degradation is important for DOX degradation. The results showed that in the non-sterile group, the degradation rate of DOX reached 77–85% on the 33rd day, while the degradation rate of DOX in the sterilization treatment group reached just over 50% on the 33rd day. Analysis of variance showed that the DOX degradation half-life in the sterilization group was longer than that in the non-sterile treatment, indicating that microbial degradation was important for DOX degradation. Wu et al. (2009) studied the microbial degradation of waste slag containing DOX at room temperature and in dark conditions and found that the concentration of DOX in the sterilization group changed little during the whole experimental period and that the degradation rate of DOX in the non-sterile group was much higher than sterilization group, which is the same with the results of our research. We also found that in the non-sterile groups, the degradation half-life of DOX in group A1 was significantly shorter than that A2 (*P* < 0.05), which was significantly shorter than that in groups A3 and A4 (*P* < 0.01). This finding demonstrated that the degradation rate of DOX in soil is affected by its initial concentration in soil. The lower the initial concentration, the faster is the degradation rate. The reason may be that a higher initial DOX concentration more strongly inhibits the microbial degradation of DOX in soil, and thus, the degradation of DOX is slower. We also found that the interactive effect on the initial concentration of DOX and different treatment has significant effects on the DOX degradation half-life and R^2^, which showed that microorganisms could significantly accelerate the degradation of DOX.

As seen from the changes in the total copies of ARGs, the total copies of ARGs in each experimental group showed an increase in the early prophase and a relatively stable trend in the later period, which was consistent with changes in the number of bacteria, fungi and actinomycetes, indicating that ARGs may be associated with the growth, reproduction and spread of microorganisms. At the same time, in the early stage, the diversity of the bacterial community markedly increased, which may have caused some antibiotic-resistant bacteria to adapt to DOX. A statistical analysis was performed on the changes in soil microbial resistance genes in each group during the experiment, and the total copies of ARGs in CK was found to be significantly lower than that in the groups added with swine manure, which revealed that the pig manure significantly increased the number of ARGs after it was added to the soil. Heuer et al. (2011) reviewed the origins of ARGs and concluded that the high abundance of microbial resistance genes on farmlands is mainly caused by horizontal transfer after swine manure is added to soil. The results of the present study also confirmed this view. By comparing the changes in ARGs among different groups with different concentrations of DOX, we found that the addition of DOX significantly slowed the growth of *tet*A and that a higher concentration of DOX had a more obvious inhibitory effect. Conversely, when the initial concentration of DOX was low, the inhibition of *tet*G and *tet*M was more obvious. Although the addition of DOX did not affect the change in *tet*W and *tet*X, supplementation with DOX decreased the number of *tet*W, and the decline trend was more obvious when the concentration was high. According to previous studies, a low concentration of antibiotics is more likely to promote the spread of ARGs. However, if the concentration is too high, it will inhibit the growth and reproduction of microorganisms and reduce the possibility of HGT (Fan and He, 2011), and we got the similar results. The addition of DOX did not increase the copy number of *tet*X, but the inhibition effect was more obvious when the concentration of DOX was high.

We also found that the addition of DOX had some influence on the change in soil microbial number and bacterial community structure and that as the concentration of DOX changed, the degree of influence also varied. The results showed that on the 33rd day, different groups had significant difference on total copies of bacteria in soil. The number of soil bacteria in the A0 group was the highest (*P* < 0.05). This finding indicates that the addition of DOX to soil significantly inhibited the number of soil bacteria and that the addition of DOX at high concentrations had an even more evident effect. It is reported that if the concentration of tetracycline in soil is higher, the inhibitory effect on the number of soil bacteria is stronger. When the concentration of added DOX was low, it showed an inhibitory effect on fungal growth. However, when the concentration of DOX was raised, it increased the growth of fungi in soil. This results corroborate the results of Thiele-Bruhn and Beck (2005), who found that oxytetracycline significantly reduced soil microbial biomass, but the number of fungi increased with increasing oxytetracycline concentrations. The possible reason is that fungi can rapidly multiply and protect themselves through spore morphology under conditions of severe and overly high antibiotic concentrations and stress and may induce the generation of resistance genes and show antibiotic resistance. When the concentration of antibiotics decreases, they can continue to be normally metabolized. Yang et al. (2009) found that tetracycline antibiotics inhibit the growth of soil bacteria and actinomycetes, significantly reducing the soil microbial number but increasing the number of soil fungi. However, in our research, the reason why the number of fungi of the low-concentration group was significantly inhibited may be that DOX concentration of the low concentration group is lower than that used in other studies. The low concentration of DOX had little effect on the number of bacteria and may have also promoted the generation of resistant bacteria so that the growth of fungi was relatively inhibited.

The present experiments showed that the addition of DOX could inhibit the growth of *Bacteroidetes*, and promote *Proteobacteria* growth, indicating that *Bacteroides* may be more sensitive to DOX and that *Proteobacteria* may easily survive in a DOX environment. Adding pig manure to the soil greatly decreased the relative abundance of *Proteobacteria* on the 0th day and increased *Firmicutes* and *Bacteroidetes*. As the experiment progressed, *Firmicutes* gradually decreased, while *Proteobacteria* and *Bacteroidetes* abundance increased significantly. This finding indicates that the addition of pig manure can change the bacterial community structure and increase the abundance of *Firmicutes* and *Bacteroidetes*, decreasing *Proteobacteria*.

Among *Proteobacteria*, at the genus level, *Pedomierobium* spp. have the highest correlation with other bacteria. *Pedomicrobium* inhabit in aquatic and terrestrial environments (Sly et al., 1988a,b). *Pedomicrobium* is known to have an ability to oxidize Mn. The highest correlation may be related to the level of Mn. *Rhodanobacter* is a Gram-negative bacterium with complete denitrification capacity (Prakash et al., 2012). Nitrate concentration is inversely proportional to pH (Spain and Lee, 2011). High nitrate concentration inhibit most microorganisms, but *R. denitrificans* can thrive. Thus, pH is the most important factor for *Rhodanobacter* (Green et al., 2012). *Sphingomonas* is a strictly aerobic bacterium. *Sphingomonas* has been found in different environments, such as terrestrial habitats and aqueous (both fresh- and sea-water), clinical specimens and plant root systems. The widespread distribution of *Sphingomonas* in the environment is due to the ability of utilizing various organic compounds and thus grow and survive under low-nutrient conditions. So *Sphingomonas* spp. could survive under harsh conditions.

Among *Firmicutes, Enterococci* is Gram-positive cocci. These bacteria have the ability to convert carbohydrates into fermentation metabolism of lactic acid. *E. faecalis* also has cation homeostasis mechanisms, it may contribute to its resistance to pH, salt, metals, and desiccation. *Enterococci* are usually catalase negative and survive in a high-EC environment. Thus, as the experiment progressed, those bacteria that can survive at high EC account for a large proportion. *Vagococcus* is a catalase-negative cocci that can survive in a high-EC environment. Studies have shown that *Caldicoprobacter* is a thermophilic bacteria; when the temperature rises, it may compete with other microorganisms and become the dominant flora (Bouanane-Darenfed et al., 2011).

One of the most active hydrolytic enzymes, urease, is important for soil nitrogen cycling. Microbial biomass, TC, and TN have positive correlation with its activity (Lyu et al., 2015). Catalase is an important enzyme in biological metabolic processes, as well as soil fauna and plant root secretion, which is of great significance for the study of soil function (Bandick and Dick, 1999). We found that the urease activity of groups A3 and A4 was significantly lower than A0, and on the 8th day A1, A2 had no significant difference to A0. On the 21st day, the catalase activity of group A4 was significantly lower than that A0–A3, which indicates that if the concentration of the initial DOX in the soil is higher, the inhibition of catalase and urease activity is stronger. At the same time, it can be seen from this study that a high concentration of DOX significantly inhibited the urease activity urease activity in some of the earlier phase of the experiment and that the inhibitory effect on catalase activity was more obvious in the latter part of the experiment.

Many studies have noted that there may be some interaction between antibiotics and soil microorganisms. For example, the abundance of ARGs is related to antibiotic concentration, residual time, etc. The change in the number of ARGs may be associated with low concentrations of antibiotics as signaling molecules for cellular communication (Takano, 2006; Struss et al., 2012). The number of soil microbes, structure and function are affected by different concentrations of DOX to a certain extent, which may mediate the occurrence of microbial community symbiosis and transfer ARGs at a high frequency (especially under low concentrations of veterinary drugs) and thus cause a large number of resistant bacteria. These bacteria are generally resistant to degradation. Once antibiotic-resistant bacteria use antibiotics as a carbon source for hydrolysis or other forms of degradation, the DOX microbial degradation process has been completed. This process is not due to the action of the soil microorganism itself but due to the interaction and alternation between the soil microorganism and DOX.

## Conclusion

In conclusion, the addition of different concentrations of DOX can affect the number of soil microorganisms, community structure and function, and at the same time, the soil microorganisms can also show some adaptability to the environment, generate DOX resistance genes, and thus survive in adversity and become dominant bacterial groups. Furthermore, some of these bacterial species show microbial degradation characteristics and play the most crucial role in the degradation of DOX.

## Ethics Statement

The experimental design and procedures followed the institutional guidelines for the care and use of animals, and all experimental procedures involving animals were approved by the Animal Experimental Committee of South China Agricultural University (SYXK2014-0136).

## Author Contributions

YW and XyL conceived the study. XyL and QY contributed to the methodology. XyL, QY, and JM contributed to the data curation. XL, BM, and YZ contributed to the investigation. JL, YW, and XdL supervised the study. QY and XyL wrote the original draft of the manuscript. QY, JM, and YW wrote, reviewed and edited the manuscript.

## Conflict of Interest Statement

The authors declare that the research was conducted in the absence of any commercial or financial relationships that could be construed as a potential conflict of interest.
