# Supplementary material for: Different Concentrations of Doxycycline in Swine Manure Affect the Microbiome and Degradation of Doxycycline Residue in Soil
Source: Front Microbiol. 2018 Dec 19;9:3129. doi: 10.3389/fmicb.2018.03129 (PMC6306040; doi:10.3389/fmicb.2018.03129)
Supplement: Supplementary file 1 [file Data_Sheet_1.docx]

**Supplementary for Material and method**

The feed method: They were fed an antibiotic-free diet for 28 days, and the feed was prepared carefully to ensure that there was no contamination of any other medicine and additives. According to the Commission of Chinese Veterinary Pharmacopoeia, 2005, DOX is allowed to be added into the daily ration at the concentration of 60-120 mg/kg daily ration. The swine were completely randomly housed (n = 12) into the following five groups: the control group that continued on antibiotic-free feed, group-1 that was fed a diet containing DOX at 30 g/t, group-2 that was fed a diet containing DOX at 60 g/t, group-3 and group-4 that was fed a diet containing DOX at 90g/t, 120g/t respectively. All these diets were given for 5 days. During the 5 days, approximately 2 kg of the manure excreted from the pigs in each group was collected twice a day.

The soil culture method: A total of 4.5 kg of soil was placed in a 5L open beaker, and deionized water was added to adjust the moisture content of the soil to 40% of the maximum field capacity. The beaker was then placed in a biochemical incubator at 23.2 ± 1°C with intermittent light (12 h light and 12 h dark) for a week to stabilize fermentation before the test. On the first day of the experimental period, depending on the designated treatment, the manure remained a certain concentration of DOX were mixed with soil and then added to the reactor, along with deionized water, to achieve a moisture content of 50% of the maximum field capacity of the soil; the soil was thereafter adjusted every 2 days to maintain this moisture content.

The method for extracting DOX and LC-MS conditions: A total of 2 g of the pig manure was placed in a 50-mL polypropylene centrifuge tube with 4 mL 0.01 Na2EDTA- McllVaine. We then performed an ultrasonic extraction for 15 min after it was whirlpool blended. This step was followed by centrifugation at 13, 000 rpm for 10 min at 4°C. The supernatants from the above process were pooled in new 50-mL polypropylene centrifuge tubes, and the above steps were repeated. Double supernatants were collected after centrifuging at 13, 000 rpm for 10 min at 4°C. Then, they were vacuum suctioned by a pump suction filter. The filtrate was transferred to a new beaker and prepared for the next solid phase extraction. A solid phase extraction column was activated with 1 mL of methanol and balanced with 1 mL of water. Before using 3 mL ultrapure water to clean the column, the filtrate was passed through the column. After remaining empty for 1 min, 1 mL methanol and 2% formic acid were used to clean the antibiotic absorbed on the column. The eluent was diluted 50 times. It was then filtered through a 0.22-μm organic membrane before testing.

The extraction was automatically injected into a **LC-MS system (**Agilent Technologies, 1200-G6410B, 5301 Stevens Creek Blvd, Santa Clara, CA 95051, USA) for analysis. If not analyzed immediately, the extracts were stored at −25 °C until analysis (no later than 1 week after the extraction). The column used in the analysis of the veterinary drug was an Agilent XDB-C18 chromatographic column (150 × 4.6 mm, 3.50 μm) at 35°C. The injection volume was 5 μL. The mobile phase was formic acid methanol. The flow rate was set to 0.40 mL/min. Tandem mass spectrometry was operated in the positive ionization mode (ESI+). The capillary voltage temperature was set to 300 °C. The gas flow rate was 9 L/min. The voltage of the atomizer was 40 psi.

The primers used for ARGs were as follows: 5’-CAGGCAGGTGGATGAGGAA-3’ and 5’-GGCAGGCAGAGCAAGTAGAG-3’ for *tet*A with a 174-bp product, 5’-GCAGAGCAGGTCGCTGG-3’ and 5’-CCYGCAAGAGAAGCCAGAAG-3’ for *tet*G with a 134-bp product, 5’-ACAGAAAGCTTATTATATAAC-3’ and 5’-TGGCGTGTCTATGATGTTCAC-3’ for *tet*M with a 171-bp product, 5’-GAGAGCCTGCTATATGCCAGC-3’ and 5’-GGGCGTATCCACAATGTTAAC-3’ for *tet*W with a 168-bp product, 5’-GAAAGAGACAACGACCGAGAG-3’ and 5’-ACACCCATTGGTAAGGCTAAG-3’ for *tet*X with a 131-bp product, 5’-ATTACCGCGGCTGCTGG-3’ and 5’-CCTACGGGAGGCAGCAG-3’ for bacteria with a 200-bp product, 5’-TCCGCAGGTTCACCTACGGA-3’ and 5’-GAGGCAATAACAGGTCTG -3’ for fungi with a 340-bp product and 5’-GGATGAGCCCGCGGCCTA -3’ and 5’- CCGCGGCTGCTGGCACGTA -3’ for actinomycetes with a 270-bp product.

**Figure S1. PCoA based on bacterial community in different groups.**


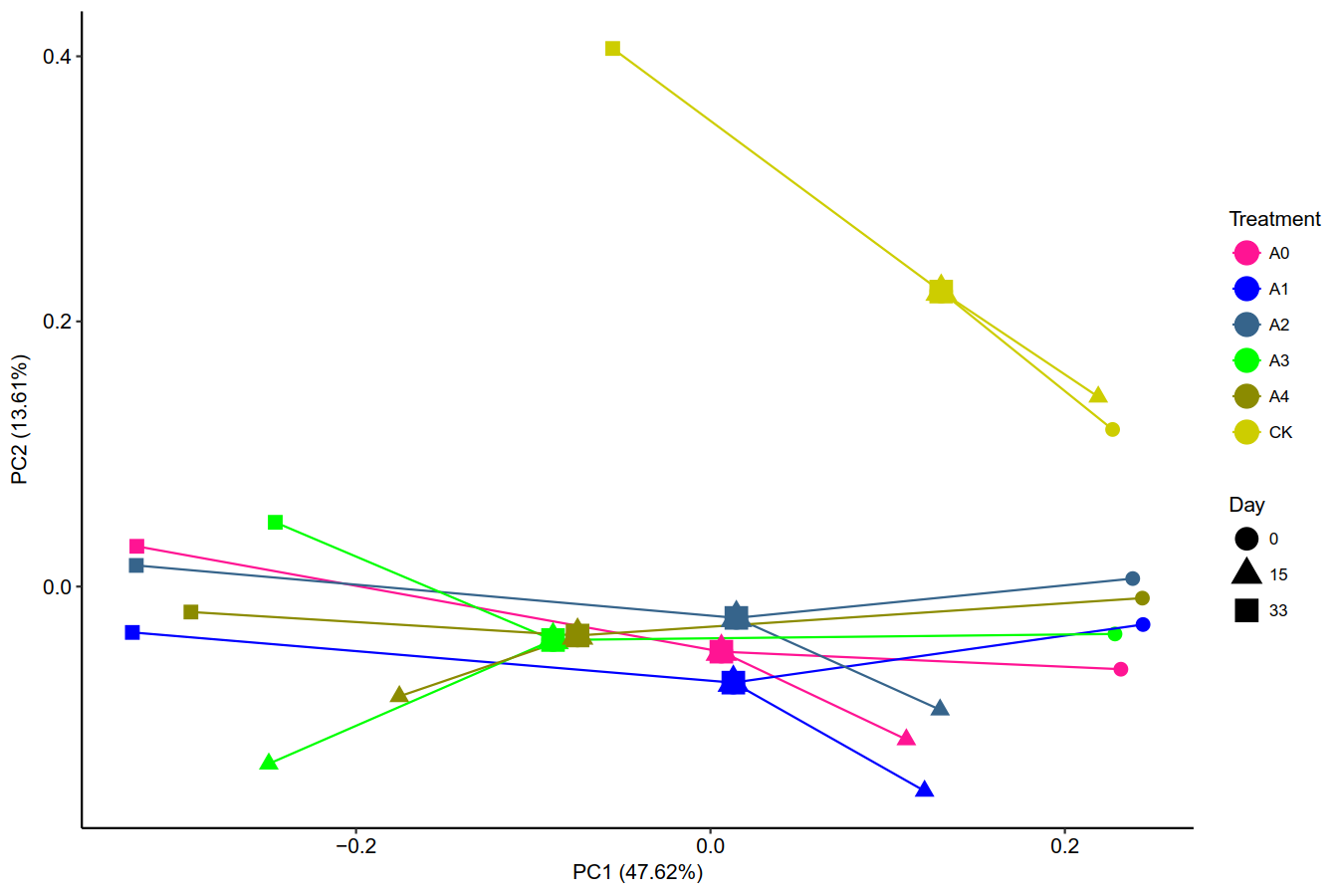


**Figure S2. Venn diagrams of various OTU levels at different times**

**Note: a, b, c are the OTU level Venn diagrams of each group at 0d, 15d, and 33d respectively.**


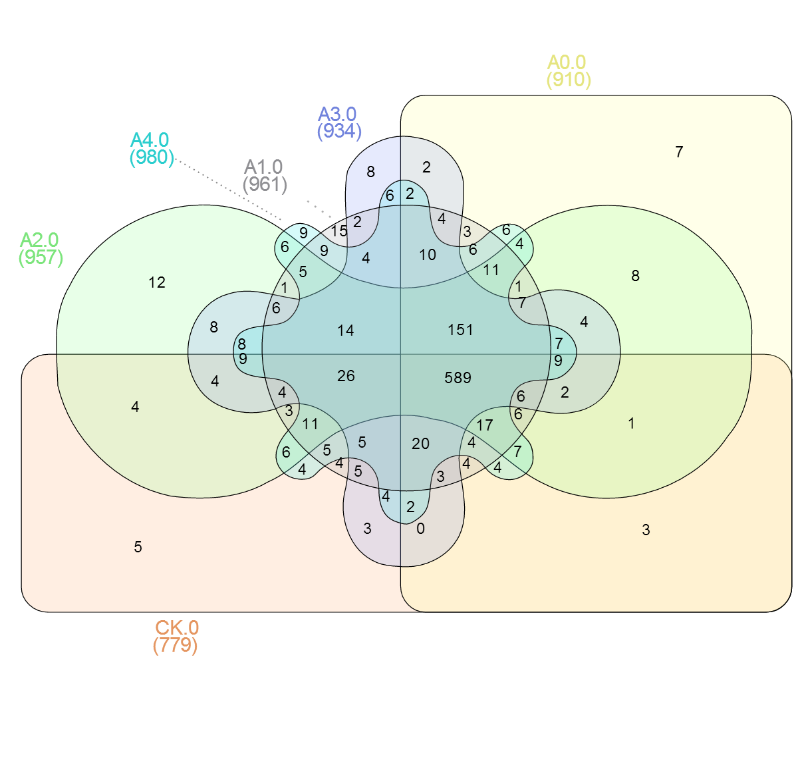


(a)


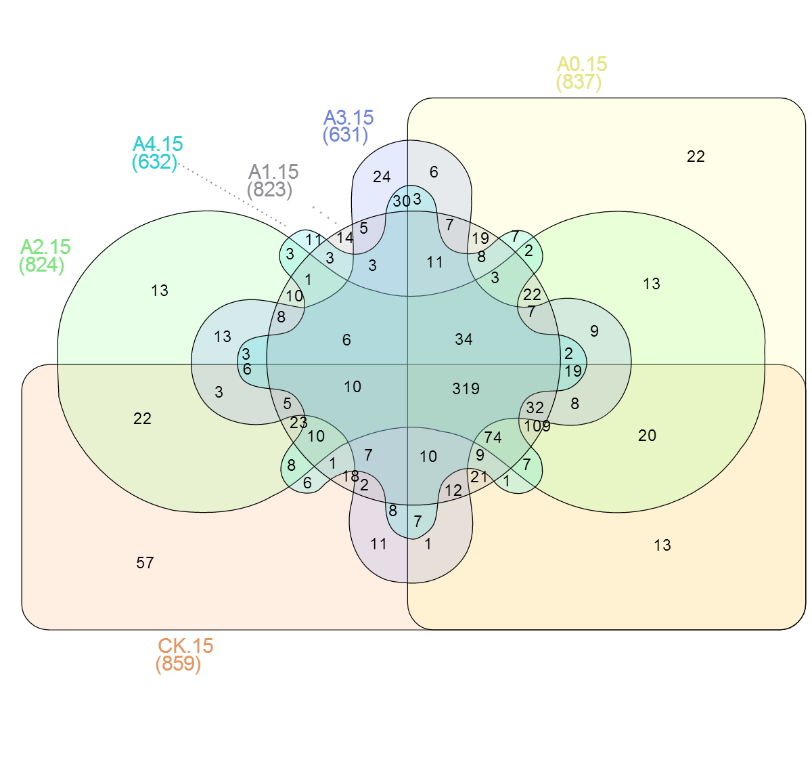


(b)


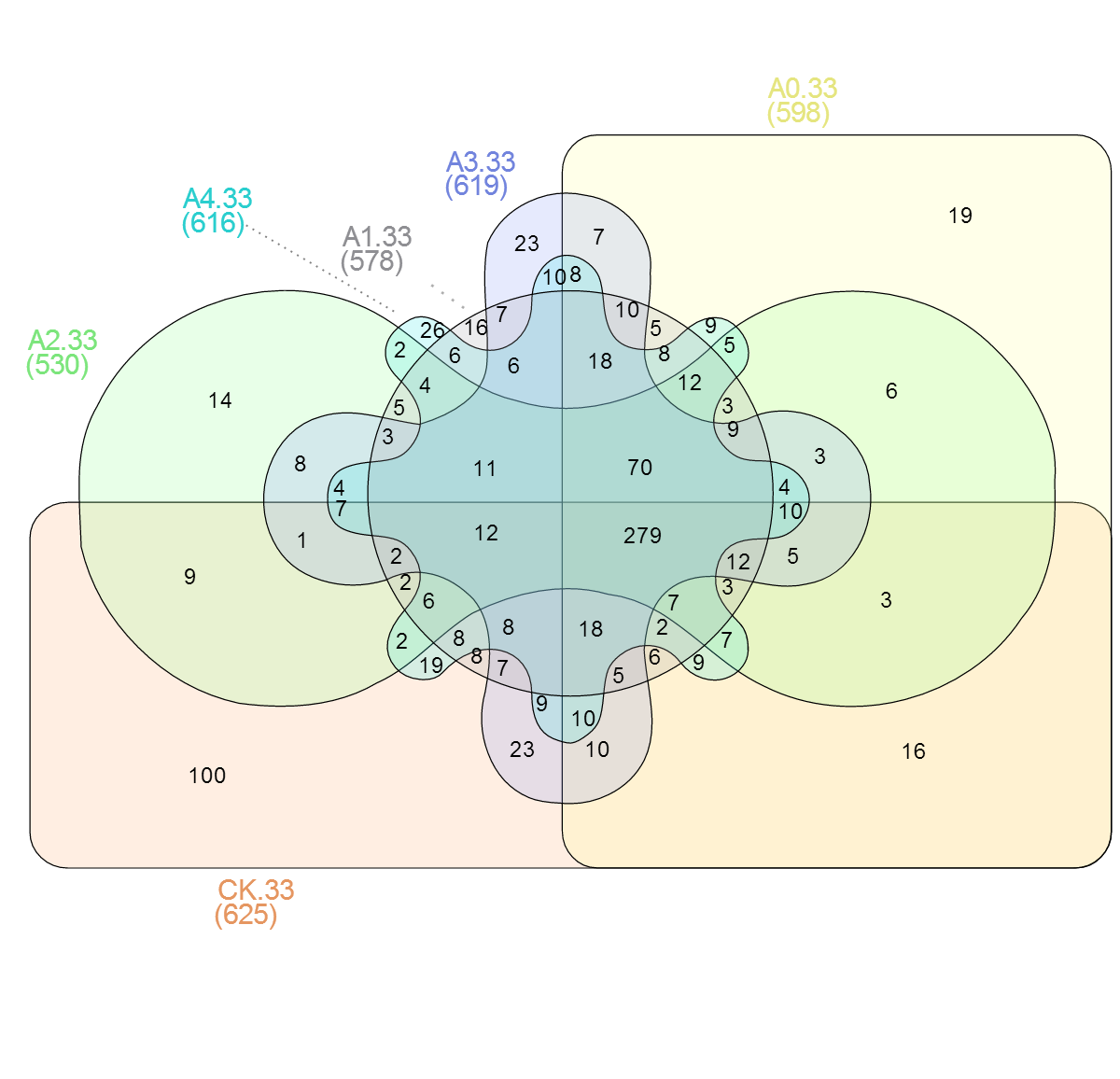


(c)

**Figure S3. The network among different genus.**


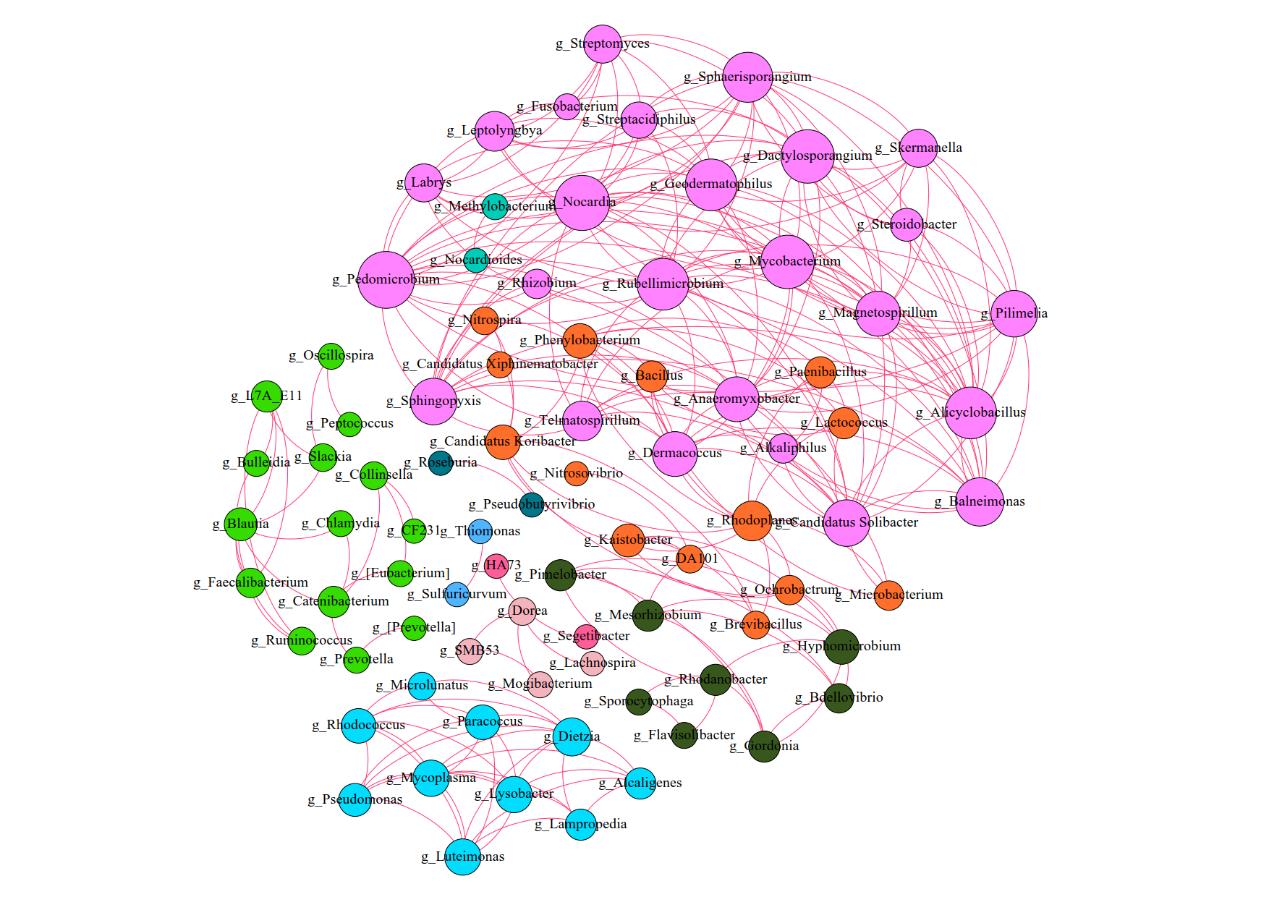


**Figure S4. Changes in the relative abundances of predominant genera under different soil physical and chemical indicators.**


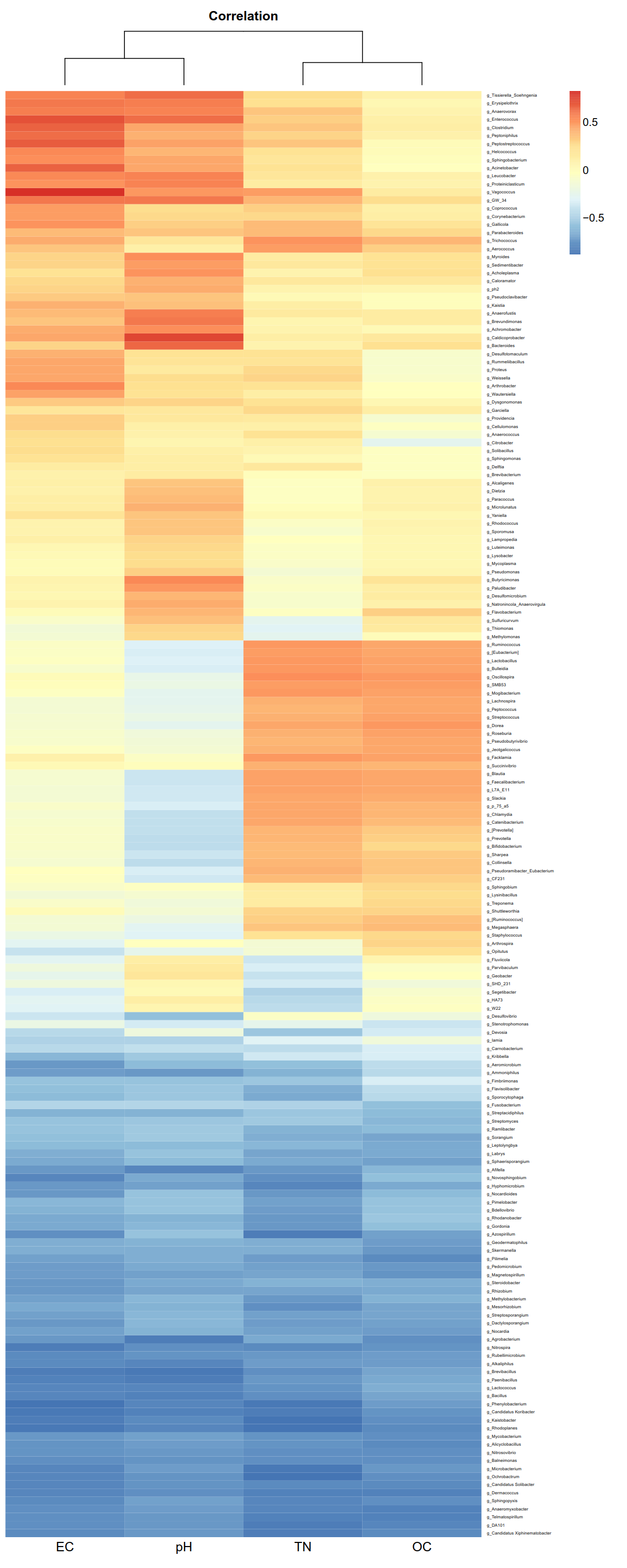


**Table S1 chemical properties of the manure from each group**

| Group | DOX  (mg·kg^-1^ DM) | pH | EC  (μs·cm^-1^) | Total carbon  (g·kg^-1^ DM) | Total nitrogen  (g·kg^-1^ DM) |
| --- | --- | --- | --- | --- | --- |
| DOX-free manure (CK) | 0 | 6.64 | 12.31 | 689.9 | 35.09 |
| Manure from swine fed with DOX (1) | 60.10±0.51 | 6.68 | 12.59 | 694.3 | 37.18 |
| Manure from swine fed with DOX (2) | 114.67±0.40 | 6.88 | 14.17 | 729.3 | 40.26 |
| Manure from swine fed with DOX (3) | 160.46±2.45 | 6.87 | 13.73 | 733.1 | 35.06 |
| Manure from swine fed with DOX (4) | 205.30±0.79 | 6.59 | 11.36 | 752.5 | 39.97 |

**Table S2 Experiment group**

| Treatment | Group |  | DOX (mg/kg) |
| --- | --- | --- | --- |
| Non-sterilization (A) | Soil mixed manure fed with diet containing DOX (A0) |  | 0 |
|  | Soil mixed manure fed with diet containing DOX (A1) |  | 4.4 |
|  | Soil mixed manure fed with diet containing DOX (A2) |  | 8.0 |
|  | Soil mixed manure fed with diet containing DOX (A3) |  | 10.9 |
|  | Soil mixed manure fed with diet containing DOX (A4) |  | 13.2 |
| Sterilization (B) | Soil mixed manure fed with diet containing DOX (B0) |  | 0 |
|  | Soil mixed manure fed with diet containing DOX (B1) |  | 4.4 |
|  | Soil mixed manure fed with diet containing DOX (B2) |  | 8.0 |
|  | Soil mixed manure fed with diet containing DOX (B3) |  | 10.9 |
|  | Soil mixed manure fed with diet containing DOX (B4) |  | 13.2 |

**Table S3 Mass spec parameters for DOX**

| Test substance | Parent ion | ion | Cone voltage（V） | Collision energy（V） |
| --- | --- | --- | --- | --- |
| DOX | 445.2 | 428.3 | 40 | 20 |
|  |  | 153.9 | 34 | 38 |
|  |  |  |  |  |

**Table S4. The temperature procedure of the conventional PCR**

| Steps | Bacteria | Fungi/  Actinomycetes | *tet*A | *tet*G | *tet*M | *tet*W | *tet*X |
| --- | --- | --- | --- | --- | --- | --- | --- |
| 1.Pre-denaturation | 94°C，240s | 94°C，300s | 94°C，600s | 94°C，600s | 94°C，600s | 94°C，600s | 94°C，600s |
| 2.Degeneration | 94°C，30s | 94°C，60s | 94°C，40s | 94°C，40s | 94°C，40s | 94°C，40s | 94°C，40s |
| 3.Annealing | 63°C，30s | 53°C，60s | 58℃，30s | 68℃，30s | 55℃，30s | 64℃，30s | 57℃，30s |
| 4.Extend | 72°C，72s | 72°C，60s | 72°C，30s | 72°C，30s | 72°C，30s | 72°C，30s | 72°C，30s |
| 5.Cycles | 35 | 35 | 40 | 40 | 40 | 40 | 40 |
| 6.Extend | 72°C，600s | 72°C，600s | 72°C，600s | 72°C，600s | 72°C，600s | 72°C，600s | 72°C，600s |
| 7.Preserve | 4°C | 4°C | 4°C | 4°C | 4°C | 4°C | 4°C |

**Table S5. Real-time Quantitative PCR thermocycling protocol for microbial and ARGs.**

| Steps | Thermocycling protocol |
| --- | --- |
| 1. Pre-denaturation | 95℃，2min |
| 2. Degeneration | 95℃，15s |
| 3. Annealing | Shown in Table S4，30s |
| 4. Extend | 72℃，30s |
| Cycles (2-4 steps) | 40 |
| 5. Dissolution curve | By default |
